# Transient Abnormal Myelopoiesis: An Abnormal Course and the Efficacy of Delayed Treatment

**DOI:** 10.7759/cureus.54219

**Published:** 2024-02-14

**Authors:** Purbasha Mishra, Mohamed Fajrudheen, Tanushree Sahoo, Tapas Kumar Som, Sandhya Biswal, Gaurav Chhabra

**Affiliations:** 1 Neonatology, All India Institute of Medical Sciences, Bhubaneswar, Bhubaneswar, IND; 2 Pediatrics, All India Institute of Medical Sciences, Bhubaneswar, Bhubaneswar, IND; 3 Pathology and Laboratory Medicine, All India Institute of Medical Sciences, Bhubaneswar, Bhubaneswar, IND; 4 Pathology, All India Institute of Medical Sciences, Bhubaneswar, Bhubaneswar, IND

**Keywords:** abnormal hematological parameters, chemotherapy, down's syndrome, neonate, transient abnormal myelopoiesis

## Abstract

Transient abnormal myelopoiesis (TAM) is observed in a few neonates with Down syndrome. While a large proportion undergo complete remission without any treatment, some of them can develop myeloid leukemia of Down syndrome (ML-DS) in the future. Without proper treatment, mortality can be high. Here we have described an interesting and difficult-to-treat case of a neonatal with Down syndrome who presented with anemia, thrombocytopenia, and 75% blasts. We came across multiple challenges in treatment due to severe pneumonia.

## Introduction

Down syndrome is the most common chromosomal anomaly affecting human beings [1] with multisystemic involvement. Children with Down syndrome have a unique predisposition to develop transient abnormal myelopoiesis (TAM). This may progress to myeloid leukemia of Down syndrome (ML-DS) in later life. Acquired N-terminal truncating mutations characterize both TAM and ML-DS in the hematopoietic transcription factor gene GATA1. Around 10-15% of children with Down syndrome have TAM during their initial days with blast >10%. Most of them undergo remission in the first three to six months. However, they require regular monitoring as the mortality approaches to 20% approximately. Children with progressive disease and features like fetal hydrops, disseminated intravascular coagulation, hyperleukocytosis (> 1 lakhs/mm^3^), and hepatopathy usually require treatment. Here we discuss an interesting case of Down’s neonate with TAM and very high blasts without life-threatening symptoms with multiple therapeutic challenges.

## Case presentation

A 2.1 kg neonate born to a 21-year-old mother with a gravida two history with no relevant antenatal aneuploidy screening was referred to us on day 24 in view of persistent oxygen requirements. In the outside hospital for sepsis with pneumonia, she was treated with IV antibiotics, oxygen and two packed red blood cells (PRBC) transfusions. On physical examination at admission, the baby had facial dysmorphology suggestive of downs phenotype with other tell-tale signs, including low set ears, flat nasal bridge, up slanting palpebral fissures, epicanthal fold, sandal gap, and generalized hypotonia suggestive of Down syndrome. On examination, she had pallor with respiratory distress.

**Figure 1:**
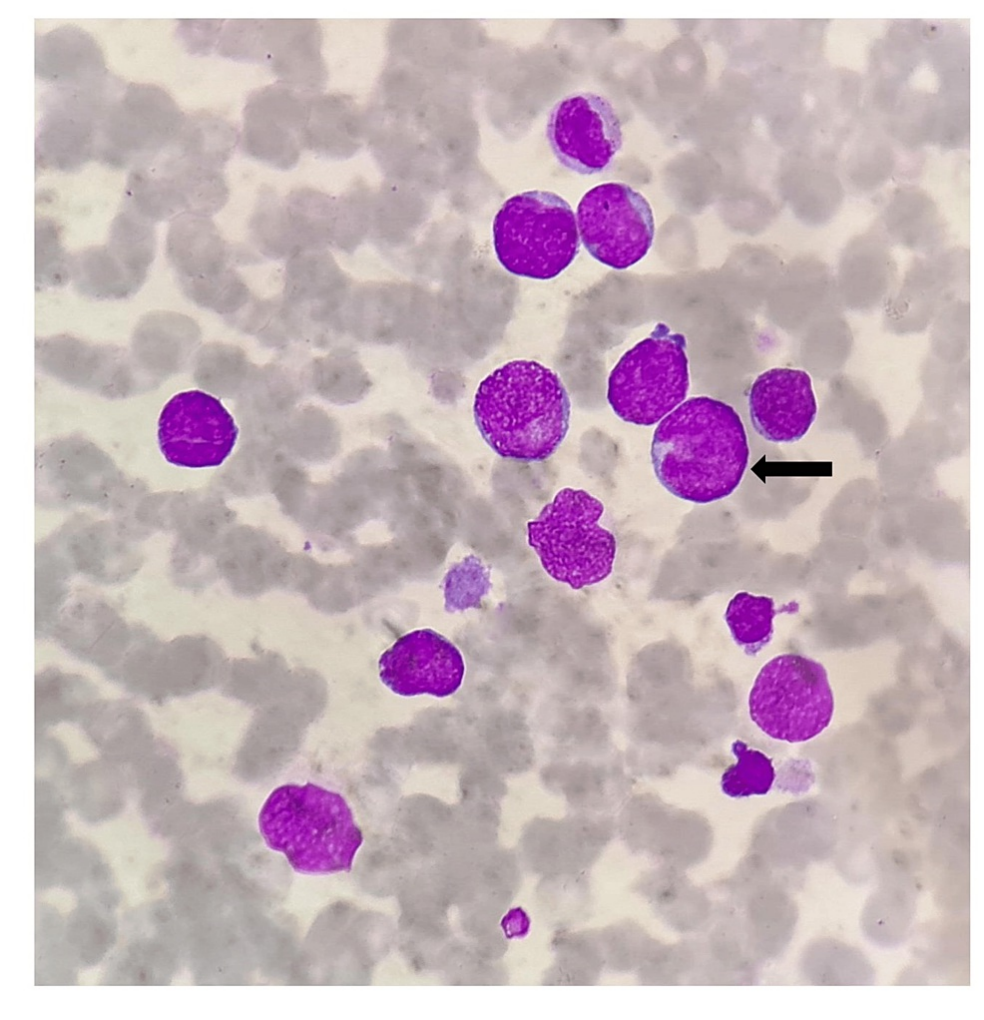
Peripheral smear showing megakaryoblast (black arrow)

**Figure 2:**
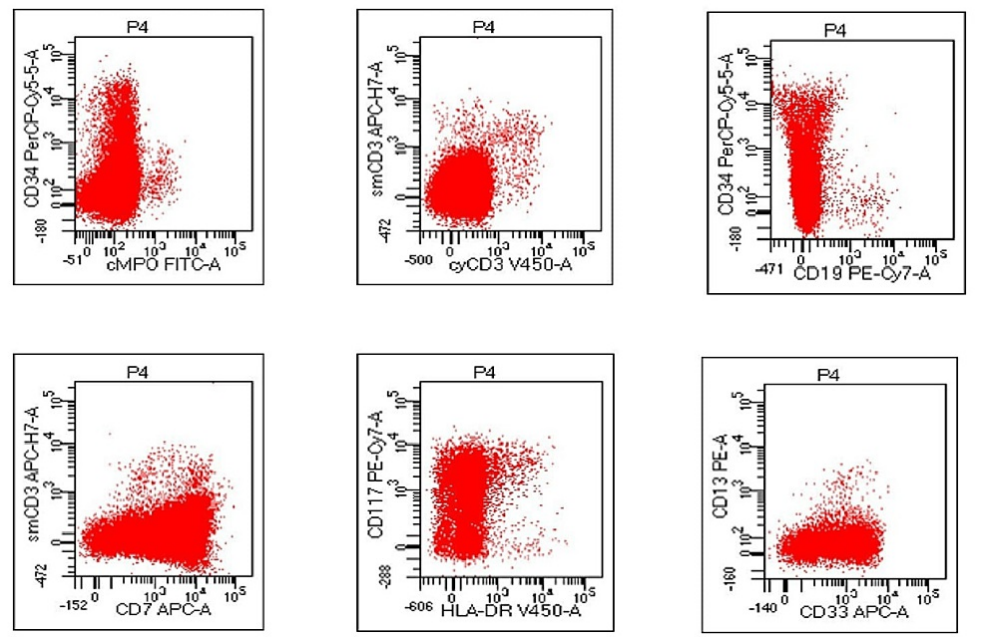
Flow cytometry showing positive for cluster of differentiation (CD) 34, CD7, CD 117 and negative for myeloperoxidase (MPO), human leukocyte antigen (HLA)-DR, CD3, CD19 suggestive for transient abnormal myelopoiesis.

Routine investigations revealed anemia (hematocrit: 23%), thrombocytopenia (19000/mm^3^), leucocytosis (86200/mm^3^), and coagulopathy (International Normalized Ratio 3). Peripheral smear showed 75% blasts with megakaryoblastic origin with flow cytometry positivity for CD 34, CD 7, CD 71, CD 41, CD 56, CD 33, CD 58, CD 117 and negativity for MPO, CD3, CD19, CD 64, CD14, CD 13, CD 66c, CD 10, CD 20, CD 79a, human leukocyte antigen - DR isotype (HLA-DR) suggestive of TAM (Figure 1, Figure 2). Chest X-ray showed right lower lobe consolidation.

Subsequently, karyotyping of index cases was confirmatory of Down syndrome (47, XX, +21).

**Figure 3:**
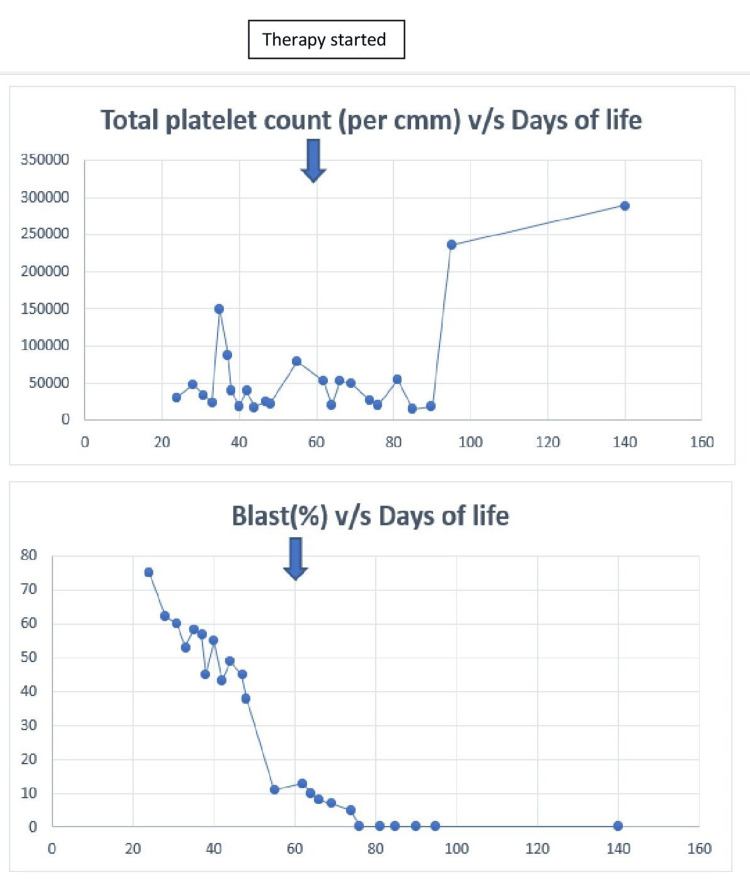
Trend showing change in platelet count and blast count with chemotherapy

At our hospital, she received supportive care in the form of mechanical ventilation, antibiotics, and blood transfusions. Due to acute respiratory distress syndrome (ARDS) and pneumonia, the infant needed mechanical ventilation on day 41. She received a fresh frozen plasma (FFP) transfusion for coagulopathy which improved with therapy. With supportive care, the blast percentage gradually reduced to 10% by day 60 of life. However, due to persistent thrombocytopenia and anemia along with blast cells remaining above 10%, seven days IV cytarabine (1mg/kg) was administered from the day of life (DOL) 60-66. Following chemotherapy, blasts were undetectable in the peripheral smear, and platelet count improved (Figure 3).

A mutation study by Sanger sequencing was negative for GATA1 mutation. The infant was extubated successfully following a prolonged intubation period on day 81 and discharged on day 142. The baby was followed up monthly with complete blood counts and peripheral smear for two months after discharge. Currently, the baby is being followed every three months to look for relapse.

## Discussion

Down syndrome in the neonatal period is often associated with various hematological abnormalities like anemia, thrombocytopenia, acute myeloproliferative syndrome, acute lymphocytic leukemia, and acute myelogenous leukemia [1]. Acute myelogenous leukemia, also called ML-DS, is 150 times more common in these children, than the general population [1,2]. TAM is a unique feature in Down syndrome, where a clonal neonatal preleukaemic state precedes acute leukemia. TAM is characterized by circulating blast cells with an acquired N-terminal truncating mutation in the hematopoietic transcription factor gene GATA1 on the X chromosome. This somatic mutation at exon 2 or exon 3 of the GATA1 gene blocks differentiation of the megakaryocytic lineage beyond the megakaryoblast stage. In the index case mutation analysis was done by Sanger sequencing which has low sensitivity to detect small mutations or there was a novel mutation that could not be detected by this method. In nearly 80% of cases, these blast cells usually disappear when TAM enters into remission leading to the resolution of symptoms and signs [3].

TAM has varied clinical presentation: ranging from incidental finding (silent TAM) in an asymptomatic baby (10-25% neonates) to more disseminated forms with hepatosplenomegaly (nearly 50% cases), liver fibrosis, effusions, coagulopathy, and multiorgan dysfunction (MODS) [4,5]. The majority will resolve without treatment; thus, observation is the key element in the treatment of these children. The indications for chemotherapy are unclear: life-threatening symptoms such as hydrops fetalis, extreme leucocytosis (WBC >100 × 109/l), hepatopathy, DIC with bleeding, and renal and cardiac failure are few to count [6]. As our index case did not have these manifestations and had severe pneumonia, chemotherapy was deferred. However, as there was the persistence of blast cells as well as thrombocytopenia needing multiple transfusions ultimately the baby was treated successfully with low doses of IV cytarabine (1 mg/kg/day) as per Berlin-Frankfurt-Münster (BFM) criteria (chemo in TAM with persisting thrombocytopenia or high white cell count (>50 × 109/l))[4]. As the Children's Oncology Group (COG) criteria are based on the presence of life-threatening symptoms which was absent in our case, we used the BFM criteria to decide on therapy.

The role of chemotherapy in causing early remission and reduction of overall mortality in children without life-threatening symptoms remains a dilemma. The BFM group observed 146 patients with 28 children receiving chemotherapy. Five-year survival in the treated and untreated groups was similar [4]. On the contrary, another study by COG involving 24 cases of refractory TAM receiving higher doses of cytarabine (3.33 mg/kg/day) had a low survival rate of 51% which was attributed to the severity of the disease, and the high dose of cytarabine that caused severe myelosuppression in 96% of children [7]. Another study by Muramatsu et al. in 168 infants with TAM reported a significant improvement in one-year survival with treatment with cytarabine in neonates with extreme leucocytosis (>100 × 109/l) [8].

Though we faced a therapeutic dilemma of whether to administer chemotherapy or not, subsequent improvement of hematological parameters after low-dose cytarabine therapy suggests an early recovery in children with non-life threatening symptoms if used in low doses. Thus, BFM criteria can be used for deciding therapy if COG criteria are not fulfilled. Multicentre large-scale studies are needed to evaluate the use of cytarabine in children with non-life-threatening TAM.

## Conclusions

All babies with Down syndrome should be screened for TAM. Though the majority undergo spontaneous remission, monitoring is needed in infants to diagnose cases with persistent high blast counts and to treat symptomatic cases. If abnormal hematological parameters persist, a trial of cytarabine should be considered.
